# Ideal Cardiovascular Health Is Associated With Slowness Among Community-Dwelling Older Adults

**DOI:** 10.1093/geroni/igaa057.683

**Published:** 2020-12-16

**Authors:** Heeeun Jung, Miji Kim, Hayoung Shim, Yuri Seo, Seoyoon Jane Lee, Chang Won Won

**Affiliations:** 1 Graduate School, Kyung Hee University, Seoul, Republic of Korea; 2 College of Medicine, Kyung Hee University, Seoul, Republic of Korea; 3 Kyung Hee University, Seoul, Korea, Seoul, Republic of Korea; 4 Elderly Frailty Research Center, Kyung Hee University, Seoul, Republic of Korea; 5 Kyung Hee University Medical Center, Seoul, Korea, Seoul, Republic of Korea

## Abstract

Slowness is associated with increased disability and mortality in older people. However, the relation between ideal cardiovascular health (CVH) and slowness in community-dwelling older adults is uncertain. We examined the prevalence of ideal CVH in Korean older adults and its association with slowness in community-dwelling older adults. We analyzed 2,597 participants (mean age 76.0±3.9 years, 54.4% women) without cardiovascular disease from the Korean Frailty and Aging Cohort Study. The usual gait speed over a distance of 4 m was measured using an automatic timer, and slowness was defined as a speed <1.0 m/s. Ideal CVH was described as attainment of ideal health behaviors (no smoking, regular physical activity, ideal body mass index, and healthy diet) and optimal health factors (blood pressure, HDL-cholesterol, and glycated hemoglobin). Multiple logistic regression analysis was used to examine the association between the CVH score and slowness. Ideal CVH was present in 785(30.2%) subjects. Considering those with poor level of CVH were as the reference group, the odds ratios [OR] for slowness were 0.55 (95% confidence interval [CI], 0.39-0.77) for those with intermediate level of CVH, and 0.28 (95% CI, 0.17-0.45) for those with ideal level of CVH after adjustment for potential confounders. Among ideal CVH components, behavioral CVH score (OR 0.65, 95% CI 0.58-0.74) was significantly associated with slowness vs. the biological CVH score (OR 0.95, 95% CI 0.84-1.07). This study indicates that ideal CVH is significantly associated with a lower risk of slowness in community-dwelling older adults. A better CVH may help prevent slowness.

